# Intricacies for Posttranslational Tumor-Targeted Cytokine Gene Therapy

**DOI:** 10.1155/2013/378971

**Published:** 2013-11-27

**Authors:** Jeffry Cutrera, Denada Dibra, Arun Satelli, Xuexing Xia, Shulin Li

**Affiliations:** ^1^Department of Musculoskeletal Oncology, Shanghai Tenth People's Hospital, Tongji University School of Medicine, Shanghai 200072, China; ^2^Department of Pediatrics, UT Graduate School of Biomedical Sciences, The University of Texas MD Anderson Cancer Center, Unit 853, 1515 Holcombe Boulevard, Houston, TX 77030, USA

## Abstract

The safest and most effective cytokine therapies require the favorable accumulation of the cytokine in the tumor environment. While direct treatment into the neoplasm is ideal, systemic tumor-targeted therapies will be more feasible. Electroporation-mediated transfection of cytokine plasmid DNA including a tumor-targeting peptide-encoding sequence is one method for obtaining a tumor-targeted cytokine produced by the tumor-bearing patient's tissues. Here, the impact on efficacy of the location of targeting peptide, choice of targeting peptide, tumor histotype, and cytokine utilization are studied in multiple syngeneic murine tumor models. Within the same tumor model, the location of the targeting peptide could either improve or reduce the antitumor effect of interleukin (IL)12 gene treatments, yet in other tumor models the tumor-targeted IL12 plasmid DNAs were equally effective regardless of the peptide location. Similarly, the same targeting peptide that enhances IL12 therapies in one model fails to improve the effect of either IL15 or PF4 for inhibiting tumor growth in the same model. These interesting and sometimes contrasting results highlight both the efficacy and personalization of tumor-targeted cytokine gene therapies while exposing important aspects of these same therapies which must be considered before progressing into approved treatment options.

## 1. Introduction

Immunotherapy is one of the most promising treatment strategies for cancer and other diseases; however, several obstacles need to be overcome before immunotherapies are widely accepted in the clinics. Several cytokines and chemokines, such as interleukin (IL) 2 [1, 2], interferon (IFN) *α* [3], IL12 [4–8], IL15 [9–12], and chemokine platelet factor 4 (PF4) [13–15], are very effective for inhibiting tumor growth via immunomodulatory mechanisms in mouse models, and dozens of either active or completed clinical trials utilize cytokines alone or as an adjuvant for treating cancer [16]. However, only IL-2 and IFN*α* have been approved by the FDA for the treatment of a small subset of cancers, and these therapies are only administered systemically in recombinant protein form [17]. One strategy that may soon help improve these therapies is gene therapy, the administration of DNA which encodes for a therapeutic protein. Although not ideal for producing all types of therapeutic proteins, the increase in safety and efficacy while reducing costs makes immune gene therapies feasible [18–20].

For most immune gene therapies the gene product must be located in the tumor microenvironment to be most effective; therefore, gene products not directly produced in the tumor need to be targeted to the tumor environment. For instance, targeting IL12 to the tumor microenvironment is critical for inducing tumor-specific T cell immune responses [5, 7, 21], and using antibodies specific for the tumor antigen L19 can increase the antitumor efficacy of IL15 [22]. Indeed, hundreds of targeting motifs have been created ranging from small peptides to large multifunctional antibodies with the intentions of improving the efficacy of multiple cancer therapies; however, the success of these targeted therapies may not only rely on the expression of the targeted ligand [3, 5, 23–26].

A previous report from our lab demonstrated the strong antitumor effects of a distantly administered tumor-targeted IL12 (ttIL12) gene therapy in multiple syngeneic cancer models [5]. This strategy utilized the tumor-targeting peptide VNTANST which targets tumor-specific ectopic expression of vimentin [27]. While further investigating the antitumor potential of the ttIL12 and the diverse potential of the VNTANST peptide, several important intricacies for successfully choosing both an appropriate targeting motif and immune payload became evident. This report will expand on the critical factors which determine the efficacy of tumor-targeted immune therapies using posttranslational delivery mechanisms.

## 2. Materials and Methods

### 2.1. *In Vitro* Experiments

The 4T1, SCCVII, EMT6, B16F10, RM1, and CT26 cell lines were purchased from American Type Culture Collection (ATCC, Manassas, VA, USA), and the LLC and K7M3 cells were donated by Augusto C. Ochoa (LSU School of Medicine, New Orleans, LA, USA) and Genie Kleinerman (MD Anderson Cancer Center, Houston, TX, USA), respectively. All cells were maintained in DMEM with 10% FBS and 1% Penn/Strep (Life Technologies, Carlsbad, CA, USA) at 37°C and 5% CO2.

The IL-12, IL-15, and PF4 plasmid DNA (pDNA) were constructed as previously described [5] using the EndoFree Plasmid Preparation Kit (Qiagen, Alameda, CA, USA). *In vitro* transfections of pDNA, IFN*γ* induction assay, and IL12/IFN*γ* ELISAs were performed as previously described [5].

### 2.2. *In Vivo* Tumor Models and Treatments

All animals and procedures performed on animals followed National Institute of Health (NIH) guidelines and were approved by the Institutional Animal Care and Use Committee at the University of Texas MD Anderson Cancer Center. Six- to eight-week-old female Balb/C, C3H, and C57/Bl6 mice were purchased from the NIH (Bethesda, MD, USA). Orthotopic tumor models were created via mammary fat pad (EMT6 and 4T1), subcutaneous (B16F10 and SCCVII), or intraosseous (K7M3) inoculations. Subcutaneous injections were used to establish ectopic tumors for CT26, RM1, and LLC models. These inoculations were performed as previously described [28]. For the K7M3 orthotopic model, the primary tumor site, right tibia, was amputated prior to the first treatment to prevent tumor-burden-mandated euthanasia. The lower limb was removed at the knee joint and the wound was closed with one or two wound clips as previously described [28].

All IL12 and IL15 pDNA were delivered via intramuscular injection of 5 *μ*g in 30 *μ*L half-strength saline in the right and left rear tibialis muscles followed by percutaneous electroporation (two 20 msec, 450 V/cm pulses with a 100 msec interval) via caliper electrodes. The PF4 treatments were performed via hydrodynamic injection into the tail vein with 10 *μ*g pDNA in 1.2 mL saline delivered in 5 to 7 s. All treatments were repeated once in all experiments, and black arrows in the figures represent treatments.

### 2.3. *In Vivo* Therapeutic Analyses

The volumes, *V*, of primary tumors for all models except K7M3 were measured via calipers measuring the longest diameter, *a*, and the diameter, *b*, perpendicular to *a*, and applying the following formula: *V* = (*π*/8)∗(*a*∗*b* ^2^). To analyze lung metastasis in the 4T1, EMT6, and K7M3 models, lungs were inflated with 15% India ink and then placed into Fekete's solution for 24 h. The next day, white nodules were counted [5]. The vessel density in 4T1 tumors as portrayed in Supplementary Figure 3 available online at http://dx.doi.org/10.1155/2013/378971 was measured via immunohistochemistry with an *α*-CD31 antibody (Cat. no. 01951A, BD Biosciences, San Jose, CA, USA) using a standard frozen section staining protocol and then counting the number of CD31-positive vessels per section.

### 2.4. Statistics

A one-way ANOVA with Tukey's post hoc test was used for ELISA and lung metastases (except for Figure 3(d)), and a two-way ANOVA with Tukey's post hoc test was used to analyze tumor volume growth rates. Mantel-Cox tests were used to analyze survival. Student's *t*-tests were used to determine significance for lung metastases in Figure 3(d), vessel density in Supplementary Figure 3, and side-by-side analyses of tumor growth or metastatic development. All analyses were performed and graphs were created with GraphPad Prism for Windows (GraphPad Software, San Diego, CA, USA).

## 3. Results

### 3.1. Location of the Peptide-Coding Sequence in the IL12-Encoding Gene Plasmid Affects the Therapeutic Efficacy of ttIL12 Treatments

**Figure 1:**
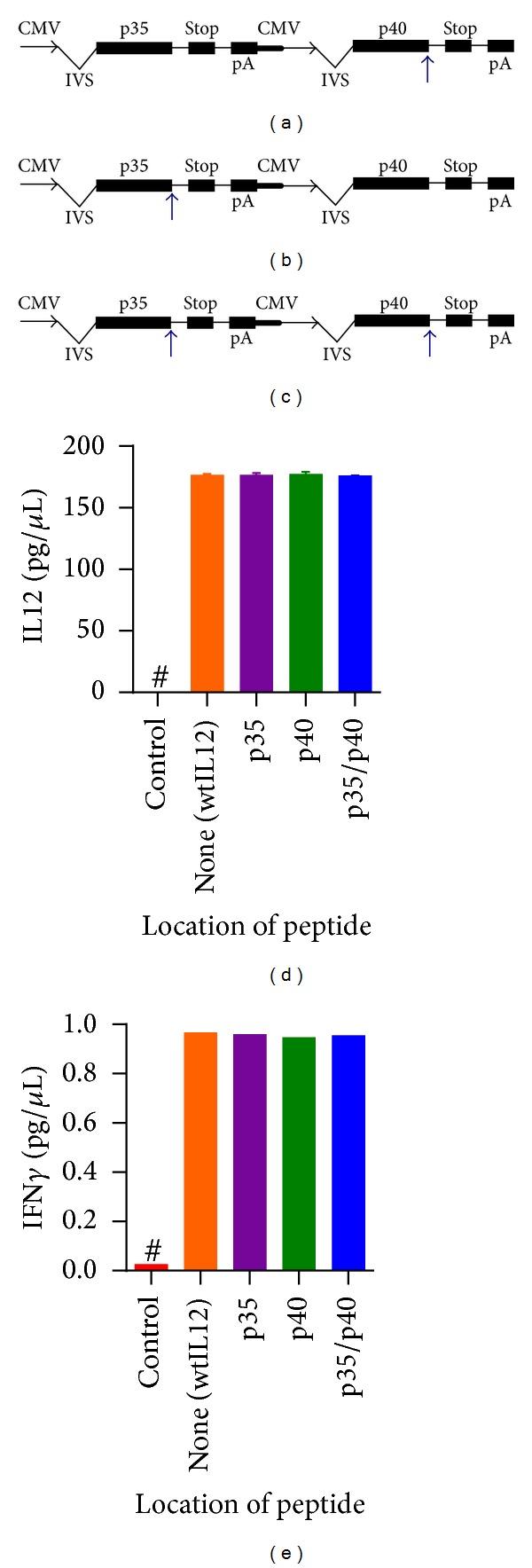
Expression and activity of IL12 is not affected by insertion of tumor-targeting sequence in either subunit. Multiple VNTANST-IL12 plasmids were created with the VNTANST sequence inserted directly prior to the stop codon in the p40 subunit (a), p35 subunit (b), or both subunits (c). The blue arrows represent the VNTANST-coding sequence insertion site in the plasmid DNA. CMV: cytomegalovirus promoter; IVS: intron; pA: bovine growth hormone polyadenylation signal; STOP: stop codon. (d) Transfection of these plasmids and wild-type IL12 and a control plasmid into cells resulted in equivalent amounts of expressed IL12 in the medium of transfected cells. (e) The peptide IL12 products induced equivalent levels of IFN*γ* from harvested murine splenocytes. # represents *P* < 0.05 compared to all other groups.

For our original experiments with ttIL12, the tumor-targeting peptide sequences were inserted directly prior to the stop codon in the p40 subunit coding region of an IL12 pDNA (Figure 1(a)). Placing the sequence in this location did not affect the expression or activity of the IL12 product [5], so the heterodimeric quaternary structure of the IL12 protein offers another potential location, the p35 subunit. Thus, two more ttIL12 plasmids were created, one with the VNTANST-coding sequence inserted prior to the stop codon in the p35 subunit (ttIL12-p35; Figure 1(b)) and the other with VNTANST-coding sequence in both subunits (ttIL12-p35/p40; Figure 1(c)). The new plasmids were capable of expressing equivalent amounts of IL12 (Figure 1(d)), and the IL12 was equally as effective for inducing IFNg from splenocytes, a hallmark of IL12 function (Figure 1(e)).

**Figure 2:**
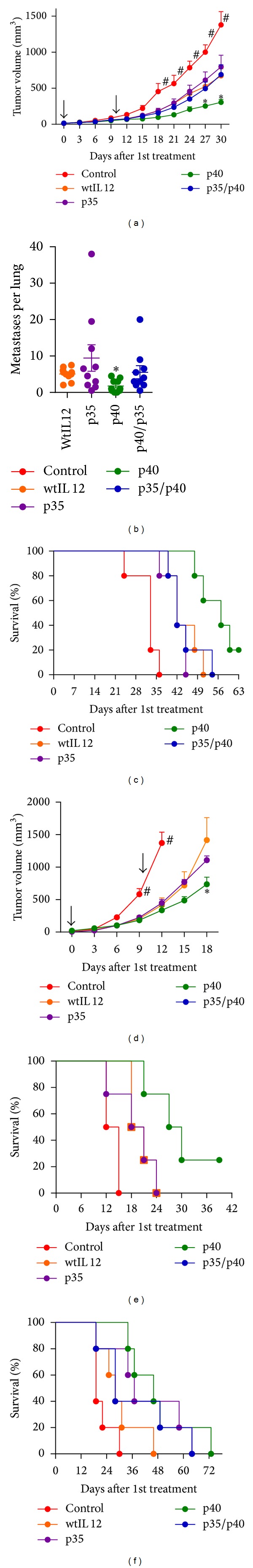
Location of the tumor-targeting sequence affects the induced antitumor response in a tumor histotype-specific manner. The ttIL12-p40 pDNA treatments increased the antitumor activity compared to the wtIL12 pDNA treatments in the 4T1 ((a) primary tumor growth; (b) lung metastases; (c) survival) and B16F10 ((d) primary tumor growth, (e) survival) tumor models. (f) In the SCCVII model, all ttIL12-peptide pDNA treatments significantly extend survival. Black arrows represent treatment dates. # represents *P* < 0.05 compared to all other groups. ∗ represents *P* < 0.05 compared to all other IL12 treatment groups.

Since the 4T1 tumor model previously responded well to ttIL12-p40 and is spontaneously metastatic, this breast adenocarcinoma model was chosen to test the efficacy of the new ttIL12 pDNAs via intratumoral injections with electroporation (EP) for treating distantly located tumors (see Section 2). Surprisingly, only treatments with ttIL12-p40 pDNA significantly inhibited primary tumor growth, reduced metastatic tumor development, and extended survival time compared to wtIL12 and other ttIL12 pDNAs, while the ttIL12-p35, ttIL12-p35/p40, and wtIL12 treatments were all effective compared to the control-treated groups (Figures 2(a)–2(c)). Differently, both the ttIL12-p40 and the ttIL12-p35/p40 significantly inhibited primary tumor growth in the colon carcinoma model CT26 (Supplementary Figure 1(a)).

Similar results were seen in the mouse melanoma model B16F10. Since no benefits were seen from the ttIL12-p35/p40 dual targeting, this group was not included in this experiment. Again, the ttIL12-p35 did not show any benefit compared to wtIL12 while the ttIL12-p40 clearly slowed the tumor progression (Figures 2(d) and 2(e)). Surprisingly, the SCCVII model produced different results. The ttIL12-p40 treatments reduced tumor volume (Supplementary Figure 1(b)) and significantly extended survival; however, there was no significant extension of survival compared to ttIL12-p35 or ttIL12-p35/p40 (Figure 2(f)).

### 3.2. The Efficacy of IL15 Gene Treatments Can Also Be Improved with the Addition of the Tumor-Targeting Peptide Sequence

**Figure 3:**
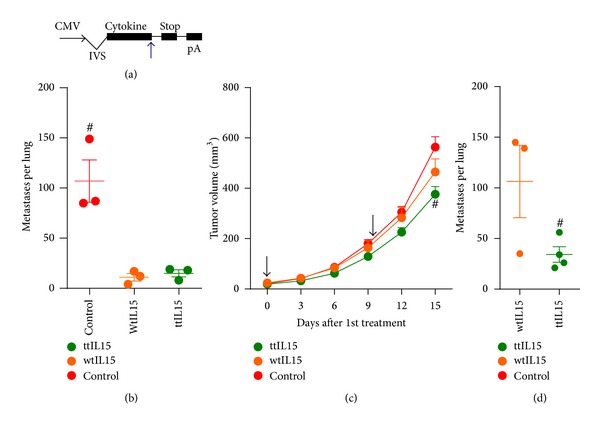
Tumor-targeting-mediated improvement of IL15-induced antitumor efficacy depends on the tumor histotype. (a) Diagrammatic representation of the IL15 plasmid with the IL15-coding region in the “Cytokine” region. The blue arrows represent the VNTANST-coding sequence insertion site in the plasmid DNA. CMV: cytomegalovirus promoter; IVS: intron; pA: bovine growth hormone polyadenylation signal; STOP: stop codon. (b) Equivalent inhibition of K7M3 lung metastases from wtIL15 and ttIL15 pDNA treatments (treatments on days 0 and 7). Inhibition of primary tumor growth (c) and metastatic lung tumor development (d) by ttIL15 pDNA compared to wtIL15 pDNA. Black arrows represent treatments. # represents *P* < 0.05 compared to all other groups.

Many other cytokines that have been employed for anticancer treatments should also benefit from targeting the cytokine to the tumor environment; therefore, a tumor-targeted IL15 (ttIL15) pDNA was constructed by inserting the VNTANST-coding sequence directly prior to the stop codon in the IL15 coding region (Figure 3(a)). First, this ttIL15 was tested in an orthotopic osteosarcoma model, K7M3. Due to the fast growing nature of the primary bone tumor in this model, the primary tumor site, the right tibia, was amputated and, therefore, only spontaneously metastatic tumors were present at the onset of treatments. Different from treatments in all other tumor models, the left and front rear tibialis muscles were the treatment sites since the right tibia was amputated. Surprisingly, the wtIL15 and ttIL15 gene treatments equally inhibited metastatic tumor growth (Figure 3(b)). Contrastingly, the tumor-targeting strategy did lead to slight inhibition of primary tumor growth in the 4T1 model. The primary 4T1 tumors in ttIL15 treated mice were significantly smaller than both wtIL15 and control-treated groups on day 15 after the first treatment (Figure 3(c)); however, the tumor growth rates were much faster than those seen with ttIL12 or wtIL12 gene treatments (Figure 2(a)). The inhibition of metastatic tumor development also increased with the ttIL15 gene treatments in the 4T1 model (Figure 3(d)), but, again, the inhibition was not as strong as seen with ttIL12 treatments (Figure 2(b)). In a separate breast cancer model, EMT6, neither wtIL15 nor ttIL15 inhibited primary tumor growth or metastatic tumor development (Supplementary Figure 2).

### 3.3. Tumor Targeting Does Not Improve Antitumor Efficacy of PF4 Treatments

**Figure 4:**
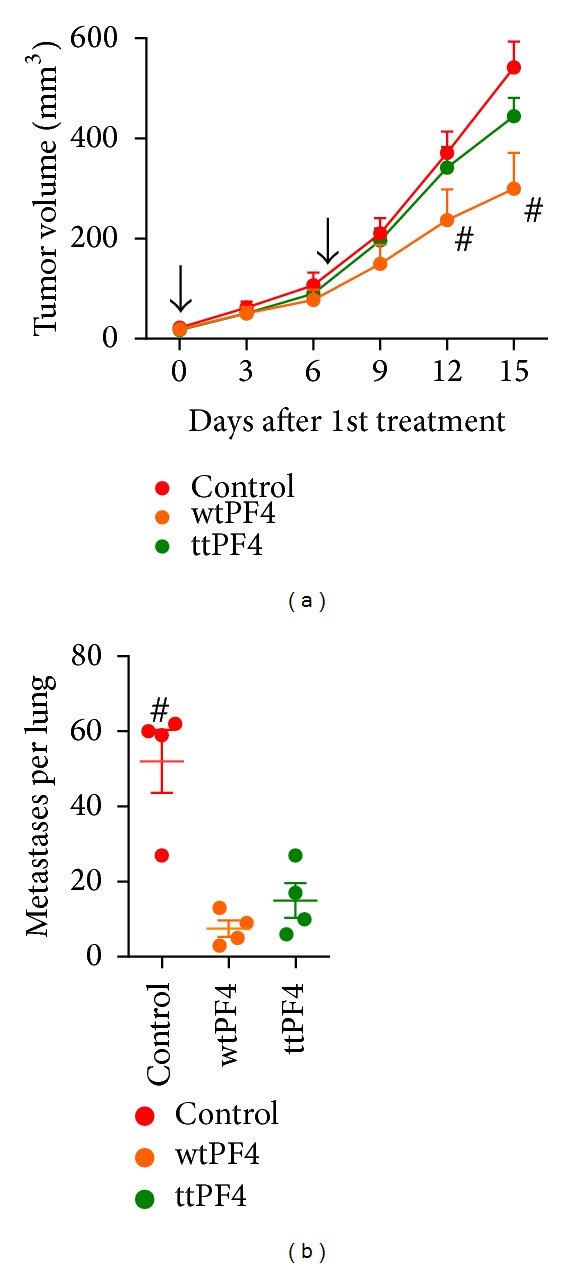
Tumor-targeting PF4 gene therapy with the VNTANST sequence does not improve anticancer efficacy. Black arrows represent treatments. # represents *P* < 0.05 compared to all other groups. (a) Only wtPF4 was capable of inhibiting primary tumor growth, but both wtPF4 and ttPF4 equally inhibited the development of lung metastases (b). Black arrows represent treatments. # represents *P* < 0.05 compared to all other groups.

Another cytokine which has shown promise for anticancer therapy is PF4. PF4 has antiangiogenic effects that can inhibit tumor growth [13]; therefore, a tumor-targeted PF4 (ttPF4) pDNA was constructed to test whether the VNTANST sequence can improve the efficacy of this antiangiogenic therapy. An *in vitro* expression assay via PF4 ELISA showed that equivalent levels of PF4 were produced from both wild-type PF4 and tt-PF4 pDNAs (data not shown). The wtPF4 treatments did show minor inhibition of primary 4T1 tumor growth, but the ttPF4 did not inhibit primary tumor growth compared to the control treatments (Figure 4(a)). Typically, PF4 and other antiangiogenic treatments can decrease the vessel density in tumors, but the vessel densities in the primary tumors were nearly identical after wtIL12 or ttIL12 treatments (Supplementary Figure 3). Interestingly, both ttPF4 and wtPF4 inhibited metastatic tumor development, but the ttPF4 treatments did not provide any further benefit for reducing the development of metastatic tumor growth (Figure 4(b)).

### 3.4. Not All Tumor-Targeting Peptides Work with this Delivery Method

**Figure 5:**
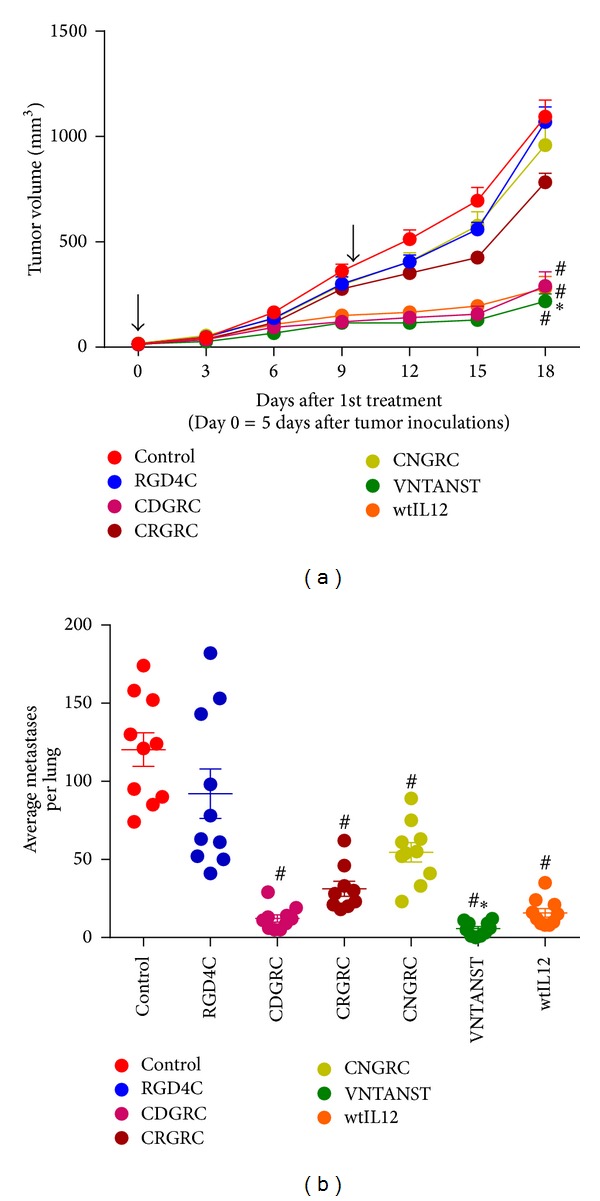
Not all tumor-targeting peptides can be successfully utilized in this gene delivery method. (a) Multiple tumor-targeting IL12 pDNAs were created by inserting the tumor-targeting peptide-coding sequences into the p40 subunit of the IL12 pDNA as in Figure 1(a). Only the wtIL12, VNTANST-IL12, and CDGRC-IL12 pDNA treatments inhibited primary tumor growth compared to the control pDNA treatments in the 4T1 tumor model. In side-by-side statistical analyses, the inhibition of primary tumor growth with VNTANST-IL12 was significantly inhibited compared to the wtIL12 and CDGRC-IL12 data on day 18 only. (b) Similar results were seen in the development of metastatic tumors in the same mice with only RGD4C not reducing development compared to control. Again, VNTANST-IL12 was significantly different from all groups in side-by-side analyses. Black arrows represent treatments. # represents *P* < 0.05 compared to control. ∗ represents *P* < 0.05 compared to all other groups.

The preceding data along with data published previously [5] has shown that the targeting peptide VNTANST can be used to increase the efficacy of IL12 and IL15 gene therapy in several tumors models. However, the efficacy of VNTANST has not been compared to other well-known tumor-targeting peptides, such as RGD4C [29] and CNGRC [30, 31]. To this end, several new tumor-targeted IL12 plasmids were constructed by inserting the coding sequences for well-documented tumor-targeting peptides prior to the stop codon on the p40-coding region (Figure 1(a)), and their antitumor efficacy was tested in the 4T1 tumor model. In this aggressive tumor model, only the targeted plasmids with VNTANST and CDGRC peptides and wtIL12 gene treatments were capable of inhibiting primary tumor growth compared to all other peptide-targeting plasmids, and only VNTANST-IL12-treated tumors were significantly smaller than wtIL12 tumors on day 18 when compared side-by-side (Figure 5(a)). Similar results were seen in an orthotopic squamous cell carcinoma model (Supplementary Figure 4). Yet, again the inhibition of metastatic tumors differs from the primary inhibition. Here, all IL12 treatments except for RGD4C-IL12 were capable of significantly inhibiting the spontaneous development of 4T1 lung metastases, and only VNTANST-IL12 treatments resulted in fewer lung tumors than wtIL12 (Figure 5(b)). Surprisingly, the RGD4C peptide, which is renowned for its tumor-targeting capabilities, did not inhibit primary or metastatic tumor growth in this strategy, but the shorter peptide CDGRC, which utilizes the same RGD targeting sequence, was able to significantly inhibit primary and metastatic tumor growth.

### 3.5. Optimal Dose of Tumor-Targeted pDNA Is Required to Achieve Successful Tumor Inhibition

**Figure 6:**
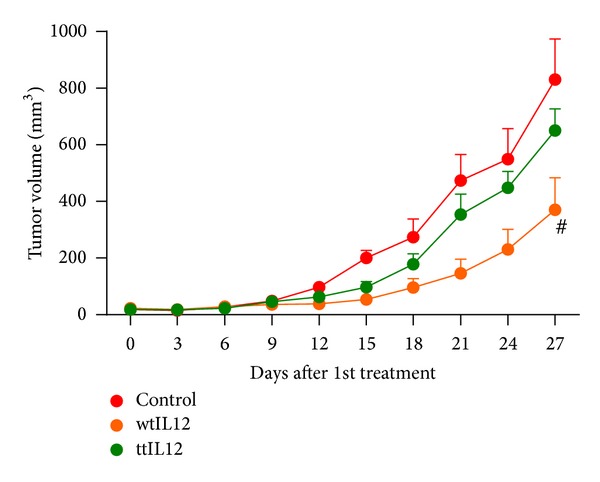
Increasing the dose of ttIL12 pDNA abrogates its therapeutic benefits. Increasing the dose by 3-fold to 30 *μ*g pDNA per treatment resulted in a loss of inhibition by ttIL12 pDNA treatments, while the wtDNA treatments retained the ability to inhibit primary tumor growth in 4T1 primary tumor growth. Black arrows represent treatments. # represents *P* < 0.05 compared to all other groups.

In an attempt to increase the efficacy of ttIL12 treatments, 4T1 tumor-bearing mice were given a higher dose, and the amount of ttIl12 pDNA administered to the mice was increased 3-fold to 15 *μ*g pDNA per rear tibialis per treatment (total of 30 *μ*g per treatment). Unexpectedly, the higher dose of ttIL12 ablated the antitumor efficacy of the ttIL12 treatments and failed to increase the efficacy of wtIL12 treatments (Figure 6).

## 4. Discussion

As cancer continues to be one of the main causes of death with little to no reduction in incidence rates, immunotherapy is on the cusp of becoming an accepted and widespread treatment option that could significantly improve the quality of life and extend survival of cancer patients [20, 21, 32–34]. However, the pleiotropic nature of cytokines and the potential for side effects necessitate that these treatments be tailored not only to the specific tumor type but also to the heterotrophic idiosyncrasies seen within each patient. The data presented in this report further displays the efficacy of the VNTANST peptide in posttranslationally tumor-targeted gene therapy while also exposing the importance of choosing the exact configuration of cytokine, targeting motif, and dose for each specific patient.

A previous publication demonstrated that the peptide VNTANST homes to tumor-specific cell-surface vimentin in breast adenocarcinoma (4T1), colon carcinoma (CT26), and squamous cell carcinoma (SCCVII) [5]. Furthermore, ttIL12 pDNA with the VNTANST-coding sequence inserted in the IL12 plasmid was more effective for inhibiting primary tumor growth, inhibiting metastatic tumor development, and extending survival in these syngeneic models. Four more tumor models were tested to determine if the ttIL12 would be effective in treating more tumor varieties. The ttIL12 only increased the antitumor efficacy of IL12 gene treatments in the melanoma model B16F10 (Figures 2(d) and 2(e)) while there were no increases in efficacy in the RM1 and EMT6 models (Supplementary Figures 5(a) and 5(b)) and a loss of any efficacy in the LLC model (Supplementary Figure 5(c)). The lack of efficacy in these models is surprising as the level of cell-surface vimentin in the LLC model is equivalent to SCCVII and CT26 models and expression in RM1 and EMT6 is 3–5-fold higher than expression in the 4T1 model (Supplementary Figure 6). These results highlight the fact that not every immune therapy will be effective for all tumor histotypes, even if the targeted motif is expressed in the tumor. So, the effects of these immunomodulatory treatments, especially those that rely on tumor-specific ligands, need to be thoroughly studied.

The different strategies for targeting immune agents to the tumor environment are continuously being tweaked and modified to improve the efficacy of the targeting. The peptide sequences RGD and NGR target to *α*_v_ *β*_3_ and aminopeptidase n [23, 29–31, 35], respectively; however, these and other peptide sequences are rarely used in their bare form [35–42]. For instance, RGD and NGR peptides are more effective when capped on each side with cysteine residues to form a cyclic secondary structure. The VNTANST targeting sequence does not contain innate self-binding cysteine residues, and modifying the sequence to include capping cysteine residues does not significantly affect the targeting abilities of the peptide (data not shown). Other studies show that adding 4 more peptides to form the RGD4C peptide (ACDCRGDCFCG) improves by 20–30-fold the affinity for its *α*_v_ *β*_3_ ligand [23, 31, 35]. Furthermore, a tetrameric RGD4C “raft” has been successfully used for imaging tumors using positron emission tomography and other imaging techniques. This and other multimeric formulations have greater affinity for the *α*_v_ *β*_3_ integrin which makes it an ideal choice for these specific imaging studies [43, 44].

On the other hand, the CDGRC-IL12 pDNA, and other peptide-IL12 pDNA, greatly surpassed the efficacy of RGD4C-IL12 gene treatments for inhibiting tumor growth in this specific gene delivery method (Figure 5(a)). In concert with these results, previously published data demonstrated that CDGRC was capable of improving the therapeutic efficacy of another therapeutic cytokine gene, IFN*α*. Structural analysis of the CDGRC peptide found that it binds not only the *α*_v_ *β*_3_ but also aminopeptidase n, the target for the NGR peptides [3]. To our knowledge, no published evidence shows that RGD4C is capable of also binding to aminopeptidase n, suggesting that the improved therapeutic efficacy of CDGRC-IL12 may be due to binding to dual targets. An alternative possibility is that the larger size of the RGD4C (1149.34 Da) peptide elicited an efficacy-depleting immune response from the host compared to the shorter CDGRC (552.64 Da) or VNTANST (705.72 Da) peptides [45]. Although the RGD4C peptide and its multimeric derivatives are undoubtedly capable of targeting payloads to tumor environments, the smaller peptides seem to be more suited to this posttranslationally targeted gene therapy strategy.

As shown previously, inserting the tumor-targeting peptide sequence prior to the stop codon in the subunit coding regions of the IL12 pDNA does not affect the expression or activity of the resulting IL12 protein. Since IL12 has 2 subunits, there may be more optimal configurations for placing the peptide sequence instead of just the p40 subunit as used for all previous experiments [5], so two other ttIL12 pDNAs were created (Figures 1(a)–1(c)). Interestingly, the effect of the peptide location appeared to be tumor-specific. In the 4T1 model, only the ttIL12-p40 pDNA was able to improve efficacy over the wtIL12 pDNA (Figures 2(a)–2(c)). Similar results were seen in the B16F10 models (Figures 2(d)-2(e)). In the less aggressive CT26 colon carcinoma model both the ttIL12-p40 and ttIL12-p40/p35 pDNAs equally improved the inhibition of primary tumors, while the ttIL12-p35 failed to improve upon the wtIL12 pDNA treatments (Supplementary Figure 1(a)). Notably, treatment with any ttIL12 pDNA extended survival in the SCCVII model (Figure 2(f)); however, only the ttIL12-p40 significantly increased primary tumor growth inhibition (Supplementary Figure 1(b)). These opposing results further advocate for the power of personalizing the targeting strategy in these therapies depending on the tumor histotype as the same targeting motif had different results based on location in different tumor models.

In addition to modifying the peptide or other targeting motif, selecting the proper immune modulatory element is critical for successful treatment of cancer. Using the VNTANST peptide to target IL15 in the 4T1 model produced interesting results. First, there was a slight yet significant inhibition in tumor growth (Figure 3(c)) and a 3-fold decrease in lung metastases compared to the wtIL15 (Figure 3(d)). However, both the primary and metastatic growth was much higher than seen with the tt- and wtIL12 pDNA treatments (Figures 2(a) and 2(b)). Contrastingly, both wtIL15 and ttIL15 equally inhibited metastatic tumor growth in the K7M3 osteosarcoma model (Figure 3(a)). Once more, the tumor histotype is an important factor to consider when choosing the targeting motif.

Additionally, the reduced effect of the IL15 could be explained by recent elucidation of IL15 in cancer therapy. Reports have shown that IL15 is in its most active form when bound to IL15r*α*, a soluble portion of the IL15 receptor [22]. So, after treatment with ttIL15 or wtIL15, the accumulated IL15 protein in the tumor environment is limited by the lack of available IL15r*α*. Although IL15 itself can upregulate the expression of IL15r*α*, a pDNA encoding for the ttIL15 with the IL15r*α* may increase the efficacy.

Targeting PF4, another cytokine which has shown some promise in anticancer therapies, was also attempted in the 4T1 tumor model; however, only the wtPF4 was able to inhibit primary tumor growth, although only slightly (Figure 4(a)). Also, both wtPF and ttPF4 were capable of inhibiting metastatic tumor growth though no differences were seen between wtPF4 and ttPF4 treatments (Figure 4(b)). Several studies have shown that PF4 can inhibit tumor growth through its antiangiogenic and immune stimulatory properties [13–15], and the slight regression from wtPF4 treatments is in agreement with other antiangiogenic treatments. However, it is surprising to see that there was no further benefit in the 4T1 metastatic tumors since the VNTANST targeting should have increased the intratumoral level of PF4, yet thettPF4 did not reduce vessel density in the primary tumors (Supplementary Figure 3).

Similarly, administering an increased amount of ttIL12 pDNA did not increase the efficacy of ttIL12. Instead, ttIL12 lost efficacy when the dose was elevated to 30 *μ*g per treatment (3-fold higher than the dose used in all other studies) in the 4T1 model, yet the wtIL12 efficacy remained the same (Figure 6). Since there were no gross signs of IL12-induced toxicity, the best dose is not necessarily the maximum tolerated dose. These results further demonstrate the importance of understanding the intricate nature of immune modulatory therapies so they can safely and effectively induce an antitumor immune response.

## 5. Conclusions

The data presented here further confirm that the VNTANST peptide is effective for delivering cytokines to both primary and metastatic tumor sites in multiple tumor models to enhance the antitumor efficacy; contrariwise, this peptide or the targeting of its payload did not improve treatment outcomes in RM1, LLC, or EMT6 models. Furthermore, this posttranslational tumor-targeting strategy can work with other tumor-targeting peptides and other cytokine payloads; however, several intricate details about these treatments must first be clearly identified. These details include the proper target in the tumor environment, the suitable targeting motif, ideal location of the motif on the payload, appropriate immune payload, and the optimal dose level, among many others. Although it may appear that these details may hinder the impact of immune gene therapies for cancer treatments, it is truly only through understanding the effects of these intricate facets of the therapy that we can develop safer, cheaper, and more effective cancer therapies.

## Supplementary Material

The Supplementary Materials contains additional data which are not necessary for the manuscript but adds to the complete story of the intricacy of these treatment strategies. Most figures expand upon the data described in the text by testing the therapies in multiple tumor models. These data show further the power and limits of this therapeutic strategy.
