# Supplementary figures and images for: Intricacies for Posttranslational Tumor-Targeted Cytokine Gene Therapy
Source: Mediators Inflamm. 2013 Nov 27;2013:378971. doi: 10.1155/2013/378971 (PMC3863455; doi:10.1155/2013/378971)

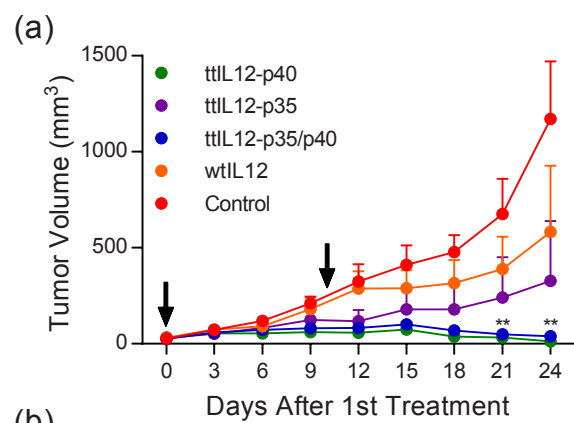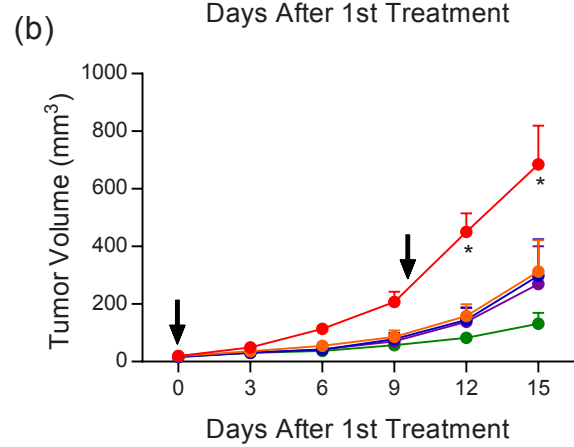

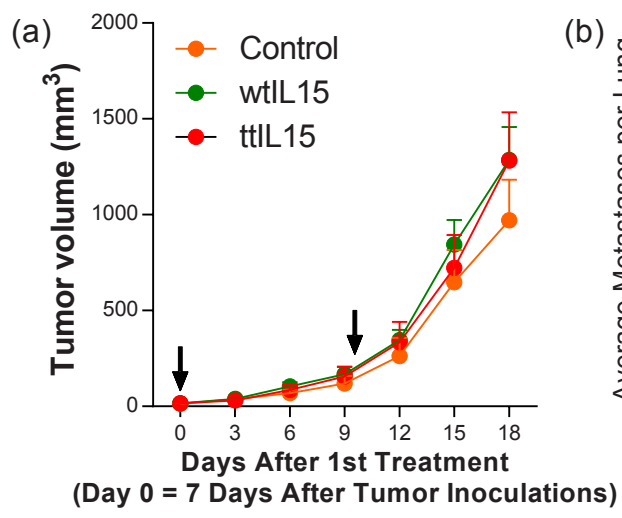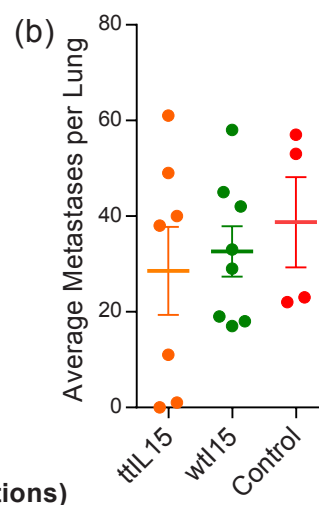

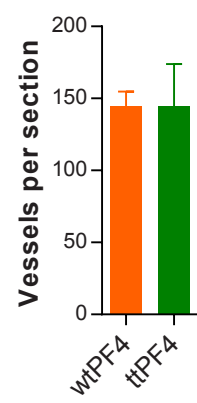

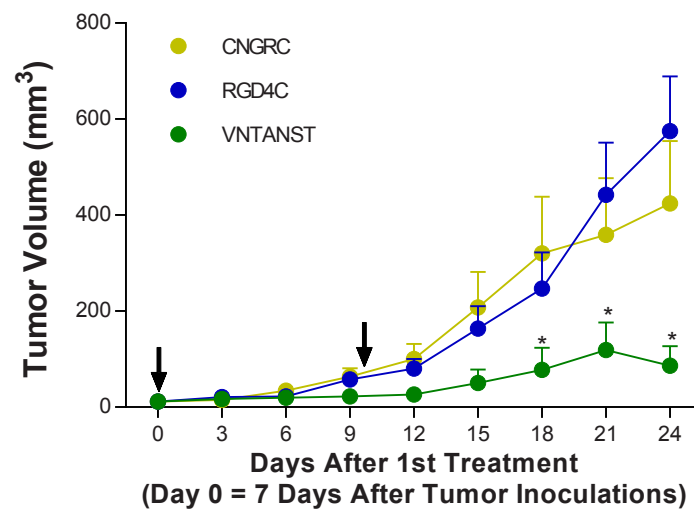

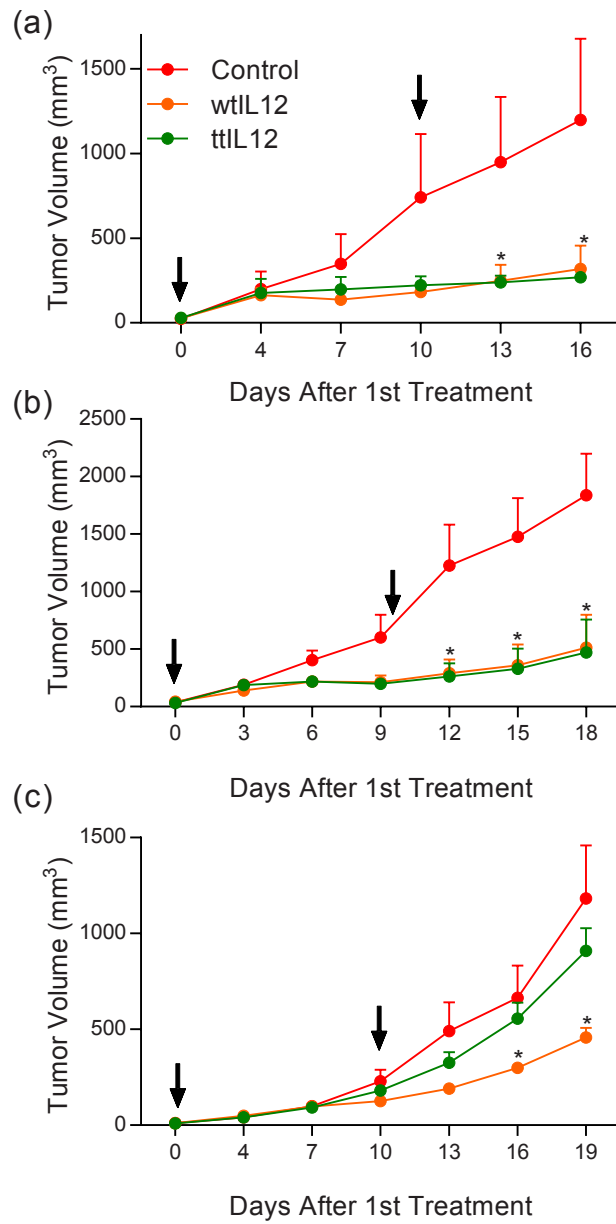

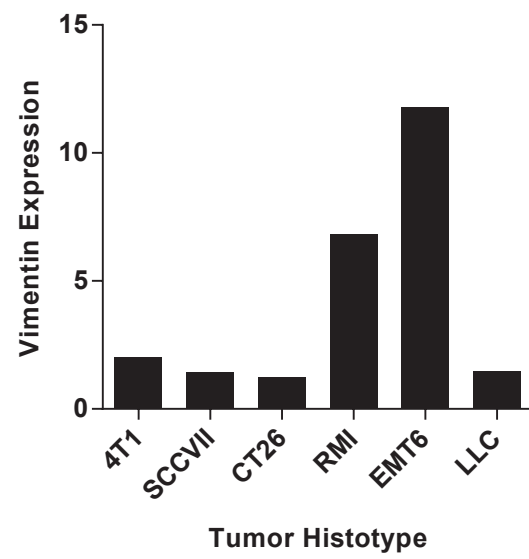

Supplement: Supplementary file 1 — The Supplementary Materials contains additional data which are not necessary for the manuscript but adds to the complete story of the intricacy of these treatment strategies. Most figures expand upon the data described in the text by testing the therapies in multiple tumor models. These data show further the power and limits of this therapeutic strategy. [file 378971.f1.pdf]
